# Associations between testosterone and future PTSD symptoms among middle age and older UK residents

**DOI:** 10.1038/s41398-025-03482-5

**Published:** 2025-08-06

**Authors:** Hanyang Shen, Ciera Stafford, Joeri Meijsen, Lijin Zhang, Jacob Reiter, Rebecca B. Lawn, Alicia K. Smith, Mytilee Vermuri, Laramie E. Duncan

**Affiliations:** 1 Department of Psychiatry and Behavioral Sciences, Stanford University, Stanford, CA USA; 2 Department of Epidemiology and Population Health, Stanford University, Stanford, CA USA; 3 Institute of Biological Psychiatry, Mental Health Center Sct. Hans, Mental Health Services Copenhagen, Roskilde, Denmark; 4 Graduate School of Education, Stanford University, Stanford, CA USA; 5 Department of Psychiatry, University of Cambridge, Cambridge, UK; 6 Department of Epidemiology at the Harvard T.H. Chan School of Public Health, Boston, MA USA; 7 School of Population and Public Health and Edwin S.H. Leong Centre for Healthy Aging, Faculty of Medicine, University of British Columbia, Vancouver, BC Canada; 8 Department of Gynecology and Obstetrics, Department of Psychiatry and Behavioral Sciences, Department of Human Genetics, Emory University, Atlanta, GA USA

**Keywords:** Psychiatric disorders, Predictive markers

## Abstract

Testosterone has been theorized to influence the development of post-traumatic stress disorder (PTSD). However, the relationship between testosterone level and PTSD is still not well understood. We evaluated the potential association between testosterone and subsequent development of PTSD symptoms using a large sample size, in a civilian context, inclusive of both males and females. Out of around 500,000 total UK Biobank participants, our sample had 130,471 participants who: had testosterone measures, completed the mental health questionnaire, and passed outlier exclusion. After adjusting for relevant covariates, we used linear regression to assess the relationship between testosterone level and future development of symptoms, in males and females separately (N_males_ = 61,758, N_females_ = 67,053). In both males and females, small but significant nonlinear (and oftentimes U-shaped) relationships were observed between testosterone levels and PTSD symptoms. When grouping participants into deciles of testosterone for both sexes, the strongest associations between testosterone levels and PTSD symptoms were observed in the central deciles. For example, for total testosterone, compared to decile 1: individuals in decile 7 had the lowest PTSD symptom scores in both males (*beta* = −0.16, *p* = 1.58 × 10^−3^) and females (*beta* = −0.23, *p* = 3.04 × 10^−5^). We also found that body mass index (BMI) moderated the relationship between testosterone and PTSD symptoms, such that the relationship was considerably stronger among individuals with higher BMI. Results were similar for depression and anxiety measures. Analyses using calculated free testosterone (cFT) and the free androgen index (FAI) were generally consistent with total testosterone (TT) results. These findings suggest that mid-range testosterone levels are associated with the lowest risk of PTSD symptoms in both sexes, and future work should seek to examine if this relationship is causal.

## Introduction

Post-traumatic stress disorder (PTSD) is a complex stress-related psychiatric disorder. Symptoms of PTSD include persistent intrusive memories, avoidance behaviors, hyperarousal, and negative alterations in cognition and mood [1]. PTSD has also been linked to increased risk of health issues such as cardiorespiratory disease, immunological disorders, and worse physical functioning [2, 3]. Moreover, comorbidity with other mental health issues such as depression and substance use is common [3]. PTSD has consistently been observed to be more common among women [4], and the past-year prevalence rates in the US are 5.2% in women and 1.8% in men [5]. Considerable studies investigating sex differences in PTSD have revealed two major categories of explanations for sex differences in PTSD: (1) social factors including trauma exposure differences and social expectations, and (2) biological factors including sex hormones and cognitive processing differences [4, 6, 7]. In the current study, we will compare the effect of an important sex hormone, testosterone, on PTSD symptoms in males and females.

The hypothalamic-pituitary-adrenal (HPA) axis is a major neuroendocrine system which regulates response to external stress [8]. Typically, when a stressor is encountered, a chain reaction known as the “HPA axis stress response cascade” is activated, releasing corticotropin-releasing hormone (CRH), adrenocorticoid hormone (ACTH), and glucocorticoids (cortisol) [8, 9]. As the brain senses the increase in cortisol, a negative feedback loop connected to the glucocorticoid receptor in the hypothalamus works to reestablish homeostasis [8]. The acute activation of the HPA response is beneficial in threatening situations, but constant activation caused by chronic stress may lead to dysregulation of this process [10], in turn contributing to psychiatric disorders, especially PTSD [8, 9, 11]. One potential explanation of dysregulation in patients with PTSD is decreased plasma cortisol levels, increased CRH activity, and increased sensitivity of the glucocorticoid receptors in PTSD patients as compared to controls [9, 11, 12], though results have been mixed. Testosterone has also been reported to interact with HPA axis activity. Viau and colleagues found higher levels of testosterone in rats were associated with restrained release of ACTH and corticosterone after stress, suggesting a suppressive effect of testosterone on stress response [13]. In human studies, experimentally increased testosterone levels have been reported to lower stress response through inhibition of CRH-released cortisol levels [14]. Thus, evidence suggests that higher testosterone could dampen stress responses, thereby reducing likelihood of developing PTSD.

Testosterone is a steroid hormone secreted by the testes in males and in much smaller quantities by the adrenal glands and ovaries in females (men have roughly 7–8, and as much as 20, times the amount of testosterone as women) [15]. While the primary function of testosterone is sexual development and maintenance of secondary sex characteristics in men, testosterone and its metabolites, e.g. estradiol and dehydroepiandrosterone (DHEA), also influence bone density, strength, and reproductive health in both sexes [16, 17]. Testosterone levels vary as a function of demographic, health, and environmental factors. Temporally, testosterone is known to vary by time of sample collection, with levels tending to be highest in the morning for both males and females [18, 19]. Over the lifespan, testosterone has been shown to decrease with age, especially in males [16]. Additionally, testosterone varies as a function of weight and body mass index (BMI); in males, higher BMI is associated with lower testosterone (negative correlation), but among females, higher BMI is associated with higher testosterone levels (positive correlation) [17, 20].

Prior studies have tested for relationships between measured testosterone in humans and PTSD symptoms [21–25]. The largest of these studies consistently reported a significant association between testosterone and future development of PTSD symptoms such that lower testosterone was correlated with higher prevalence of PTSD (Josephs, *N* = 120; Reijnen, *N* = 918) [21, 25]. Three smaller studies (sample sizes of 9, 40, 98) found no significant relationship between testosterone and PTSD [22–24]. Most prior studies were conducted in military [21–23, 25] rather than civilian [24] contexts. Of the five previous studies, four out of five included only men, and the fifth (Josephs) included 16 female soldiers. Josephs found no significant sex difference in their results, though that conclusion should be understood in the context of the small number and percentage of female participants (N_total_ = 120; N_women_ = 16). Because prior studies either included no or very few females, potential relationships between testosterone and PTSD remain largely unexplored. Finally, to our knowledge, only linear relationships between testosterone and PTSD have been investigated.

Given the evidence that testosterone may be related to PTSD through HPA axis functioning [14], the relationship between testosterone levels and future PTSD symptoms warrants investigation. Here we leveraged the UK Biobank’s large sample size to test for non-linear relationships between testosterone measures and PTSD symptoms, in both males and females. This UK Biobank dataset is the largest sample available with measures on both testosterone and PTSD symptoms; see methods for limitations of this biobank-scale measurement of PTSD symptoms.

Although in previous literature a negative correlation between testosterone and BMI was found [22], there are few studies specifically exploring the interaction effect between testosterone and BMI on health outcomes. Diaz-Arjonilla and colleagues [26] found BMI modified the inverse relationship between testosterone and erectile dysfunction with a stronger association among obese participants (i.e. higher testosterone corresponded to lower risk for erectile disfunction), and Wang and colleagues [27] found a similar BMI-modifying effect on the inverse relationship between testosterone and sexual dysfunction and cardiovascular diseases (in both instances, the “protective” effect of higher testosterone was stronger among higher-BMI individuals). Thus, appropriate testosterone levels could possibly be more important for people with higher BMI. Therefore, we also tested for a potential modifying effect of BMI on the relationship between testosterone and PTSD symptoms. We addressed these questions using a sample far larger than any used previously (>60 times the largest prior investigation), in a civilian population, and including both males and females.

## Subjects and methods

### Participants

The UK Biobank is a biomedical repository of data from around 500,000 participants from the United Kingdom (UK) with variables spanning genetic, social, imaging, medical, lifestyle, and environmental domains [28]. The baseline questions and blood samples were collected between 2006–2010. Participants were included because they were registered with the National Health Service (NHS) and lived within traveling distance to a total of 22 assessment centers across the UK. Recruitment was conducted in conjunction with the NHS via written invitations to participate that were mailed to prospective participants’ homes [29]. The NHS provided prospective participant names, addresses, and dates of birth and the invitation included a phone number participants could call with any questions or concerns [29, 30]. After pilot testing and peer reviewing of the recruitment process for clarity, informed consent, and user-friendliness of the touchscreen questionnaires was completed in 2005–2006, the first round of invitations was sent to individuals aged 40–69 in Manchester, UK in 2007. Invitations were then expanded in the UK to a broader age range in 2007–2010 [30]. In total, invitations to participate were sent to 9.2 million individuals, of which 502,150 consented to participate and attended the recruitment session, yielding an acceptance rate of 5.45%[31]. This sample was somewhat representative of the UK population at the time of measure (95% self-reported White-identifying in UKB cohort; 92% self-reported White-identifying in 2001 UK census), though may be less representative of the current UK population as the percentage of White-identifying people has decreased (82% self-reported White-identifying in 2021 census) [31]. A mental health questionnaire was sent to participants in 2016 via email, and participants who did not provide email addresses were encouraged via the annual newsletter. Our analysis included all participants who completed the mental health questionnaire (*N* = 159, 251), which included PTSD items (described below). Exclusion criteria for participants were: missing data on the mental health questionnaire, missing serum testosterone measurement from the baseline blood collection, and having values for baseline serum testosterone levels that were classified as outliers using the standard outlier definition (i.e. values >1.5 times the interquartile range, on either side of the interquartile range) [32]. Outliers were computed separately in males and females. Using the interquartile range method, most of the excluded participants had high values of total testosterone. Among males, the original range of total testosterone is 0.354–50.623, compared to the range in our analytical dataset of 2.592–21.187; among females, the original range of total testosterone is 0.350–31.424, compared to the range in our analytical dataset, 0.350–2.325. 1089 (1.7%) males and 1907 (2.7%) females were excluded due to extreme testosterone values. The final sample size was 130,471 (62,565 male and 67,906 female). For fully adjusted models (i.e. when requiring no missing covariates for any person) the sample size decreased by 1.3% to 128,811 participants (61,758 male and 67,053 female).

### PTSD phenotype

In 2016, the UK Biobank deployed a mental health questionnaire (MHQ) designed by an expert working group, expanding opportunities to explore mental health research topics. The PTSD symptom scores within the MHQ were collected using six questions modified from the PTSD Checklist for DSM, version 5 (PCL-5) [33]. It is important to note that our PTSD symptom measure overlaps with other psychiatric disorders and does not include the hallmark diagnostic criteria of PTSD, like re-experiencing symptoms or past trauma. As such, throughout the paper, we utilize the term “PTSD symptoms” to refer to this PTSD symptom score. The PTSD symptom scores have been used in multiple studies exploring PTSD risk in UK Biobank samples [33–35]. The tradeoff of a lower quality measure affords the opportunity to assess the relationship between testosterone and PTSD in this much larger sample than otherwise possible (*N* = 140,000 vs a sample size of 100 s or 1000 s with a gold standard clinical measures). Per prior use of these scores, the final list of six questions assessed the following potential PTSD symptoms as experienced in the last month: felt irritable or had angry outbursts, avoided activities because of stressful experiences, felt distant from other people, had repeated disturbing thoughts, felt upset, and had trouble concentrating. Scores for the first five questions ranged from 1–5 and scores for the last question ranged from 1–4, with higher scores indicating greater severity of the symptoms. The scores of each of the six questions are then summed to equal a value that could range from 6–29 [33, 34]. This aggregated PTSD symptom score is our primary outcome.

### Testosterone measurements

A variety of testosterone measures are commonly used in medical studies of testosterone, including total testosterone (TT), calculated free testosterone (cFT), and the free androgen index (FAI), which were used as the three major predictors of this study. Testosterone can be free circulating in the bloodstream (free testosterone) or can be bound to other metabolites, like albumin (weak bond) and sex hormone binding globulin (SHBG, strong bond) [36]; the sum of these is referred to as total testosterone (i.e. the free and bound testosterone). Total testosterone is typically measured via assay to detect testosterone in blood. In contrast, because free testosterone is a small fraction of total testosterone (an estimated 2–4%), it is often more effective to use validated mathematical inference methods to estimate free testosterone rather than measure it directly [37]. One commonly used method is calculated free testosterone (cFT), which uses measurements of albumin and SHBG levels (in addition to measured total testosterone) to calculate free testosterone [38]. Similarly, the Free Androgen Index is a calculated ratio between total testosterone and SHBG to yield an estimate of available testosterone [39]. We use all three measures, TT, cFT, and FAI, in our analyses.

Blood samples were collected from UK Biobank participants upon recruitment. Serum total testosterone (TT) levels were measured by chemiluminescent immunoassays (Beckman Coulter DXI800). Participants with total testosterone below the limit of detection were considered as missing in this study; their data were not available to us. To estimate free testosterone levels, we used the formula published by Vermeulen et al. [38], yielding calculated free testosterone (cFT) levels for all participants (see below). Sex-hormone binding globulin (SHBG) and albumin levels were measured by chemiluminescent immunoassay Beckman Coulter DXI800 and colorimetric assay Beckman Coulter AU5800, respectively. We also calculated the free androgen index (FAI): the ratio of total testosterone to SHBG multiplied by 100 [39].

Calculated free testosterone (cFT)

$${\rm{cFT}}=\frac{\sqrt{-b+\left({b}^{2}-4{ac}\right)}}{2a}$$

in which $$a=(\left[{\rm{albumin}}({\rm{g}}/{\rm{L}})\right]\times \frac{36000}{69000}+1)$$

$$b=[{\rm{SHBG}}({\rm{nmol}}/{\rm{L}})]-[{\rm{testosterone}}({\rm{nmol}}/{\rm{L}})]+(\left[{\rm{albumin}}({\rm{g}}/{\rm{L}})\right]\times \frac{36000}{69000}+1)$$

$$c=-[{\rm{testosterone}}({\rm{nmol}}/{\rm{L}})]$$

Free androgen index (FAI)

$${\rm{FAI}}=\frac{\left[{\rm{testosterone}}({\rm{nmol}}/{\rm{L}})\right]}{[{\rm{SHBG}}\left({\rm{nmol}}/{\rm{L}}\right)]}\times 100$$

### Statistical analysis

Given the sexual dimorphism of testosterone levels in males and females, we always analyzed males and females separately. To determine the optimal model for the relationship between testosterone and PTSD symptom scores, we evaluated models incorporating linear, quadratic, and cubic terms. We employed the likelihood-ratio test to determine which model offered the best fit to the data. In addition to our primary analysis in which testosterone was kept as a continuous variable, we also – for visualization purposes – divided testosterone levels into deciles; these results are presented in our figures with corresponding statistics. After outlier removal, our sample did not contain any male participants in the clinically high testosterone range; for example, 7.6% of the males in this analysis (all assigned to the first decile) had clinically low testosterone, meaning total testosterone levels below the normal range for men aged 50–59 (215–878 ng/dL) as defined in Barrett-Connor’s study [40]. The covariates used were those known to influence testosterone levels (age, time of sample collection, and body mass index) as well as standard demographic covariates: socioeconomic status as quantified in the Townsend deprivation index and ethnicity as reported in the UK Biobank. Confidence intervals were calculated from two-sided model parameters. *P*-values from Wald test were reported for regression parameters. We adjusted for multiple testing with Bonferroni-correction: *p* < 0.05/9. To explore the possible modifying effects of BMI on the relationship between testosterone and PTSD symptom variables, we then reported the stratified models by BMI categories, using Centers for Disease Control (CDC) thresholds (underweight BMI < 18.5, healthy weight BMI 18.5–24.9, overweight BMI 25–29.9, and obese BMI > 30) [41]. All analyses were conducted in R, version 4.2.2. For our sensitivity analyses and comparative analyses, we include results without removing outliers, as well as results using a proxy anxiety symptom score, the Generalized Anxiety Disorder (GAD-7) score, and a depressive symptom score, the Patient Health Questionnaire (PHQ-9), as secondary outcomes. Like the PTSD symptom score, these two scores were collected from the mental health questionnaire. GAD-7 included 7 questions, and the final score ranged from 0–21. PHQ-9 included 9 items, and the final score ranged from 0–27.

## Results

### Participant characteristics

The baseline characteristics of study participants are shown in Table 1. In this sample, the average PTSD symptom score was 7.6 in males and 8.2 in females. The prevalence of participants with PTSD symptom scores higher than 12 was 6.5% in males and 9.7% in females. The majority of participants recorded their ethnicity as “white” (97.8% in both males and females). Regarding CDC BMI categories, less than 1% of this sample was in the underweight category (BMI < 18.5).

**Table 1:** Baseline characteristics of study participants.

Variables used in the study	Males		Females	
	(*n* = 62, 565)		(*n* = 67, 906)	
	Mean/n	(SD/%)	Mean/n	(SD/%)
PTSD symptom score	7.6	(2.8)	8.2	(3.2)
Predictors				
Age at baseline (years)	56.6	(7.8)	55.0	(7.7)
Townsend deprivation index	−1.8	(2.8)	−1.7	(2.8)
Time of blood collection (24 h)	14.4	(3.0)	14.4	(2.9)
Ethnicity				
White	60,497	(97.8%)	65,703	(97.8%)
Asian	671	(1.1%)	439	(0.7%)
Black	408	(0.7%)	513	(0.8%)
Mixed	313	(0.5%)	531	(0.8%)
BMI (continuous)	27.3	(4.0)	26.4	(4.9)
BMI				
Underweight (BMI < 18.5)	116	(0.2%)	503	(0.8%)
Healthy weight (18.5< = BMI < 25)	17,820	(28.9%)	29,932	(44.6%)
Overweight (25< = BMI < 30)	30,694	(49.7%)	23,824	(35.5%)
Obesity (BMI> = 30)	13,129	(21.3%)	12,797	(19.1%)
Testosterone-related measurement				
Total testosterone (nmol/L)	11.91	(3.3)	1.05	(0.4)
total testosterone (ng/dL)	338.6	(93.5)	29.9	(12.3)
SHBG level (nmol/L)	39.1	(15.5)	63.7	(30.6)
SHBG level (ng/dL)	1111.5	(441.8)	1809.9	(868.7)
Albumin level (g/L)	45.7	(2.6)	45.1	(2.5)
Calculated free testosterone (cFT) (nmol/L)	0.2094	(0.051)	0.0128	(0.006)
cFT (ng/dL)	5.9500	(1.436)	0.3640	(0.180)
Free Androgen Index (FAI) (%)	32.11	(9.4)	1.86	(1.1)

### Measures and model selection

**Fig. 1:**
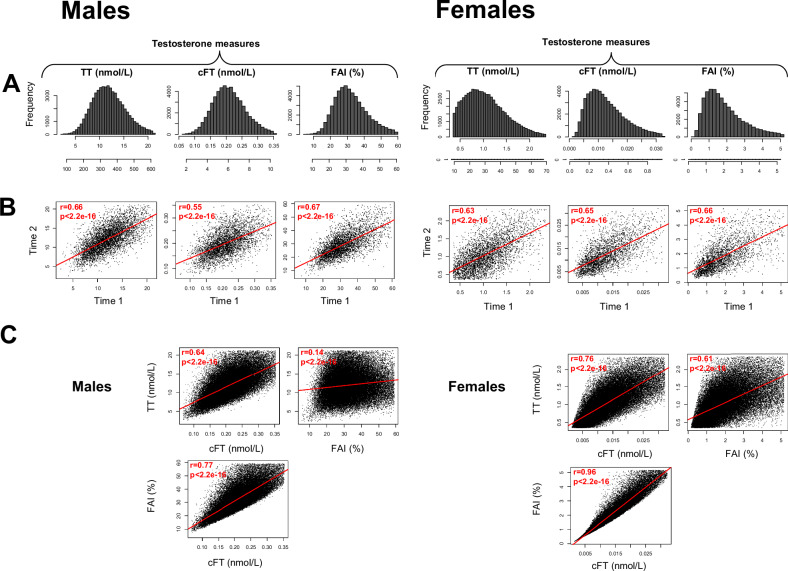
Distributions and reliability of testosterone measures, and relationships among measures. **A** Distributions of the three testosterone measures in males (left) and females (right) **B** Scatterplots of correlations between time 1 and time 2 measures of each of the testosterone measures, in males and females. Note that a second measurement of testosterone was available for a subset of the sample (*n* = 4925 males, *n* = 3817 females), and that the time between measures varied across participants from 2–7 years, and thus these values reflect lower bounds on the reliability of testosterone measures **C** Scatterplots of each testosterone measure against the others.

We first assessed the distributions and reliability of the testosterone measures used in the UK Biobank. As can be seen in Table 1, female testosterone levels (TT mean/sd nmol/L = 1.05/0.4; ng/dL = 29.9/12.3) were lower than male testosterone values (nmol/L = 11.9/3.3; ng/dL = 338.6/93.5). Fig. 1 shows the distributions of the three testosterone measures (TT, cFT, and FAI) after outlier removal. To assess the reliability of total testosterone measures in the UK Biobank, we used the subset of participants who had two measures of testosterone (*n* = 4925 in males and *n* = 3817 in females); the second measure was taken 2–7 years after the first, and therefore the correlations between time 1 and time 2 reflect lower bounds of the reliability of these testosterone measures. In both males and females, correlations were higher than 0.6, for all three testosterone measures except for cFT in males with *r* = 0.55.

Regarding correlations among the three testosterone measures after outlier removal, in the male group, the correlations between total testosterone and cFT (*r* = 0.64, *p* < 2.2 × 10^−16^) and cFT and FAI (*r* = 0.77, *p* < 2.2 × 10^−16^) were relatively high, but the correlation between total testosterone and FAI was relatively low (*r* = 0.14, *p* < 2.2 × 10^−16^), see Fig. 1C. In females, the correlations between pairs of the three testosterone measures were generally higher than those observed in males: testosterone and cFT (*r* = 0.76, *p* < 2.2 × 10^−16^), total testosterone and FAI (*r* = 0.61, *p* < 2.2 × 10^−16^), cFT and FAI (*r* = 0.96, *p* < 2.2 × 10^−16^).

In both males and females, modeling a quadratic relationship between total testosterone and PTSD symptoms (the reference model), rather than only a linear relationship, provided a significantly better fit to the data (males: *p* = 1.00 × 10^−8^ and females: *p* = 8.19 × 10^−4^), see Table 2. Modeling of a cubic relationship did not show further advantage compared to a quadratic relationship (males: *p* = 0.40 and females: *p* = 0.23). In both males and females, the decile model was nearly equivalent to the quadratic model. Thus, we consider our primary model to be the quadratic model (i.e. the one using linear and squared terms for testosterone), and we use the decile-based model to facilitate plotting of results. As described below, results were consistent using both models. Models with covariates showed the same pattern of results as the models without covariates.

**Table 2:** Comparison of model fit in predicting PTSD symptoms using linear, squared, cubic, and decile terms for testosterone (always including lower order variables), with and without covariates.

Model	Variables in Models	Males				Females			
		n	R^2^	logλ	*p*-value	n	R^2^	logλ	*p*-value
1-1	TT	62,565	0.0004	−153606	1.00E-08	67,906	0.0002	−175902	8.19E-04
1-2	TT+TT^2^	62,565	0.001	−153589	ref.	67,906	0.0003	−175897	ref.
1–3	TT+TT^2^+TT^3^	62,565	0.001	−153589	0.40	67,906	0.0004	−175896	0.23
1–4	Decile TT	62,565	0.0008	−153593	0.32	67,906	0.0004	−175895	0.73
2-1	TT + Covariates	61,631	0.0287	−150211	3.70E-05	66,923	0.029	−172122	1.09E-04
2-2	TT+TT^2^ + Covariates	61,631	0.029	−150202	ref.	66,923	0.0293	−172115	ref.
2-3	TT+TT^2^+TT^3^ + Covariates	61,631	0.029	−150202	0.42	66,923	0.0293	−172114	0.43
2–4	Decile TT + Covariates	61,631	0.0289	−150204	0.87	66,923	0.0293	−172111	0.49

Covariates were age, time of collection, BMI, ethnicity, and Townsend deprivation index.

*TT* total testosterone, *Log*$\lambda$ log-likelihood.

### Associations between testosterone and PTSD symptoms

**Fig. 2:**
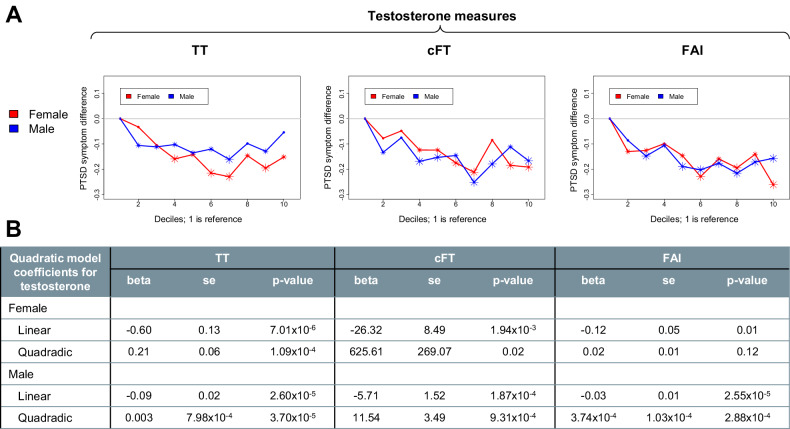
PTSD symptom difference, by sex, for deciles of testosterone measures. The first decile (i.e. 0–10% of participants) is the reference group for all tests. **A**. From left to right, results are shown for total testosterone (TT), calculated free testosterone (cFT), and free androgen index (FAI). Points depict average difference in covariate-adjusted PTSD scores for each decile compared to the first decile. Smaller asterisks indicate nominal significance (*p* < 0.05) and bigger asterisks denote Bonferroni-corrected significance. **B**. Parameter estimates are shown for quadratic models. Betas, standard errors, and *p*-values were calculated from linear regressions using both testosterone linear and quadratic terms with covariates predicting PTSD symptom scores. Covariates were age, time of sample collection, BMI, Townsend deprivation index, and ethnicity.

The associations between testosterone measures (total testosterone, cFT, and FAI) and PTSD symptoms are provided in Fig. 2A and Supplementary Table S1 (decile models). Both the quadratic and the decile models revealed non-linear relationships between testosterone measures and PTSD symptom scores. In males, when considering total testosterone, the decile model indicated the minimum risk for PTSD symptoms in decile 7 (nmol/L:12.5–13.5, ng/dL:360.5–389.4) with *beta* = −0.16. In females, the minimum risk was also at decile 7 (nmol/L: 1.1–1.2, ng/dL: 31.7–36.1), with *beta* = −0.23. The highest average PTSD symptoms were among participants in the first decile as expected, for both sexes. It is interesting to note that even in the clinical normal range of the testosterone (decile 2 to decile 10), the risk of experiencing PTSD symptoms can be quite different. In males and females, cFT and FAI showed similar patterns. For both sexes, linear and quadratic terms for testosterone measures were almost always significant predictors of PTSD symptoms (Fig. 2B). In all instances, the beta for the linear term was negative and the beta for the quadratic term was positive, indicative of a concave or U-shaped relationship between testosterone measures and PTSD symptoms. For example, in males, for total testosterone, the linear term was *beta* = −0.09 (*se* = 0.02, *p* = 2.60 × 10^−5^) and quadratic term was *beta* = 0.003 (*se* = 7.98 × 10^−4^, *p* = 3.70 × 10^−5^), and in females *beta* = −0.60 (*se* = 0.13, *p* = 7.01 × 10^−6^) and quadratic term was *beta* = 0.21 (*se* = 0.06, *p* = 1.09 × 10^−4^).

### Interaction between testosterone and body mass index (BMI) on PTSD symptoms

**Fig. 3:**
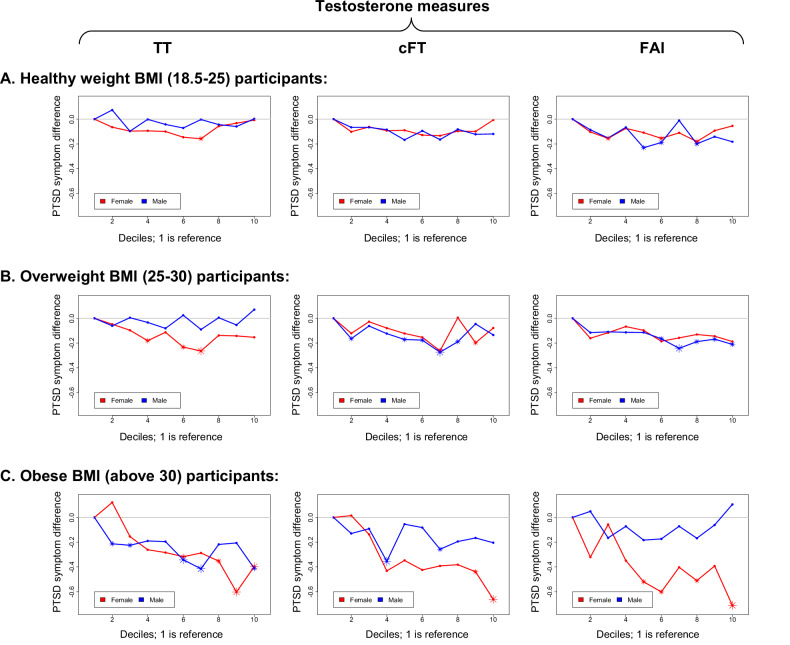
PTSD symptom difference for deciles of testosterone measures with the first as the reference, stratified by BMI category. For all plots, the y-axis is PTSD symptom score difference. From the left to the right columns, results are shown for total testosterone (TT), calculated free testosterone (cFT), and free androgen index (FAI). **A**. Results among participants with healthy weight BMI (18.5–25). **B**. Results among participants with overweight BMI (25–30). **C**. Results among participants with obese BMI (above 30). Solid circles indicate non-significant differences, smaller asterisks indicate nominal significance (*p* < 0.05), and bigger asterisks denote Bonferroni-corrected significance.

Including interaction terms for BMI and testosterone measures in quadratic models significantly improved the fit of the models (except the model for cFT in males). For example, considering total testosterone (TT), inclusion of the interaction terms (TT×BMI and TT^2^×BMI) improved fit in males (*p* = 0.0003, *df* = 2) and females (*p* = 0.0003, *df* = 2). Fig. 3 and Supplementary Table S2–S4 show the associations between testosterone measures and PTSD symptoms stratified by BMI categories. For both males and females, stronger associations between total testosterone and PTSD symptoms were observed among participants with higher BMI.

### Outlier sensitivity analysis and comparative analyses with anxiety and depression

For the analyses without removing outliers, as shown in Supplementary Figs S1–S3 and Supplementary Tables S5–S8, the correlations among the three testosterone measures were reduced. However, the overall association and BMI-stratified association between testosterone levels and PTSD symptoms are mostly the same except with increased risks for the final decile group. For quadradic models, although the coefficient changed by a relatively large amount, the direction did not change, as the betas for the linear terms remained negative and the betas for the quadratic terms remained positive. We also conducted the same analysis using GAD-7 and PHQ-9 as comparison (Supplementary Figs S4–S7 and Supplementary Tables S9–S16). Similar trends as were found in the PTSD symptom results showed for both anxiety and depression, which may indicate that the non-linear association with testosterone and the BMI-stratified effects are related to some shared factors across common psychiatric disorders.

## Discussion

The main finding of this work is that there is a highly statistically significant relationship between measured testosterone levels and future PTSD symptoms, in both males and females. Low testosterone levels, normed by sex, were associated with the highest future PTSD symptoms. Mid-range testosterone levels (deciles 3–8) were associated with the lowest future PTSD symptoms. Analyzing data from 130,471 participants, this study is over 60 times larger than the largest previous investigation of the relationship between testosterone and future PTSD symptoms, affording the testing of more nuanced models than previously investigated. In addition, whereas nearly all prior investigations excluded women, we were able to analyze the relationship in both sexes with greater statistical power than previous studies. Because this is one of the first studies with a broad time span, large sample size including both males and females, and civilian population, these results may not be directly comparable to previous studies. However, prior studies on testosterone and PTSD provide valuable context for interpretation. We also observed that the relationship between testosterone and PTSD symptoms depended on BMI, such that the relationship strengthened as BMI increased. Our results suggest that – though modest in magnitude – there is a highly significant relationship between testosterone levels and future development of PTSD symptoms, especially among individuals with higher BMI. Our findings are consistent with previous work that has shown testosterone to have anxiolytic and antidepressant effects in men, particularly in men with low testosterone [42]. Additionally, one recent genetic study showed a negative correlation between PTSD and testosterone in men which implies that for males, the genetic variation associated with higher testosterone levels were correlated with a lower genetic risk of PTSD [43]. In women, there is also some evidence that testosterone can have anxiolytic and antidepressant effects, but high levels may also lead to new onset mood episodes, consistent with the findings in our study of a U-shaped relationship between PTSD symptoms and testosterone [42].

Several biological mechanisms for testosterone’s impact on the HPA axis have been proposed in previous studies. Testosterone is believed to modulate the HPA axis either through direct actions at androgen receptors or via metabolism to other compounds that interact with androgen and estrogen receptors [10]. Both testosterone and its metabolite dihydrotestosterone (DHT) have been shown to have anxiolytic effects via actions on the androgen receptor. The testosterone metabolite 3β-diol activates estrogen receptor beta (Erβ), which has an inhibitory effect on the HPA axis. In addition, testosterone can also be metabolized to estrogen, which can have both activating and inhibitory functions on the HPA axis. While androgens are capable of producing these HPA-activating effects through their metabolites, systemic testosterone is generally thought to overall reduce stress-induced HPA responses and increase negative feedback on the HPA axis [43, 44]. Testosterone may affect corticotropic releasing factor receptors, thereby influencing HPA axis functioning [10]. Another mechanism through which higher testosterone could reduce HPA activation is through increasing the activity of oxytocin neurons in the paraventricular nucleus of the hypothalamus [8], potentially leading to inhibition of the release of corticotropin-releasing hormone (CRH) to CRH receptors.

The novel nonlinear relationship we observed between testosterone and PTSD symptoms in men and women may also be explained by indirect or competing influences of testosterone on the HPA axis and the brain. The HPA and hypothalamic–pituitary–gonadal (HPG) axes are believed to have reciprocal effects on one another, and HPA dysregulation is believed to underlie PTSD and other psychiatric disorders, including depression. The “dual hormone” hypothesis suggests that the combined profile of testosterone and cortisol levels is what affects mood and behavior rather than each hormone individually [21]. For example, there is some evidence that testosterone is related to aggressive disorders only in the presence of low cortisol [21, 45–47], but studies to date have not supported a relationship between high testosterone and low cortisol in PTSD [21]. Cortisol measures were not available in UK Biobank, so we could not explore the dual hormone effect in this study. Future studies further exploring this question should collect both testosterone and cortisol levels to differentiate the effects.

Our results also showed interaction between testosterone and body mass index on PTSD symptoms. Testosterone levels in men are inversely related to BMI [48] such that total testosterone levels are low in obese men. Despite normal free testosterone levels, centrally obese men have shown a hyperresponsiveness of the HPA axis (increased cortisol and ACTH response to stimulatory tests) [49]. In males with increased obesity, there is also increased aromatase activity, which irreversibly converts testosterone to estradiol and results in decreased testosterone and elevated estrogen levels [50] where estrogen itself may have independent effects on symptoms. In general, these findings suggest that BMI is not merely a confounder for testosterone, but also an important modifier which is consistent with our results [26, 27, 50]. Therefore, more studies exploring how BMI, and other body composition measures that more directly distinguish body fat from muscle, modify the impact of testosterone on psychiatric disorders need to be conducted to further unveil this mechanism.

There are several limitations in our study. First, the PTSD symptoms evaluated did not encompass all diagnostic criteria for PTSD. While participants were asked questions about traumatic experiences, traumatic experiences were not specifically related to PTSD symptoms, so were not included in our PTSD symptom score [51]. Furthermore, the included symptoms overlap with other psychiatric disorders, and do not assess hallmark symptoms of PTSD. The availability of data in this large biobank therefore limited our ability to specifically assess PTSD, and we cannot conclude that this relationship between testosterone and PTSD symptoms is specific to PTSD. As such, our outcome should be considered with these limitations. Second, no PTSD symptoms were measured at baseline concomitantly with testosterone levels, so it is unknown if these symptoms developed after the testosterone levels were obtained or before. Similarly, the specific timing of traumatic events was not assessed in this study, so it is unknown if the testosterone levels were obtained after or before a traumatic event. Therefore, the association reported here could be attributable to, wholly or in part, the impact of PTSD symptoms on testosterone levels. Third, one limitation of the interpretation of our results is addressing testosterone level as a risk factor for PTSD. Since our data is time-spanning, but not longitudinal, we do not fully have the advantages of a longitudinal study design. Instead of indicating that testosterone levels can be used as a clinical biomarker for PTSD, our study only suggests that testosterone levels are a potential risk factor for later PTSD symptoms. Our results could also be explained with the hypothesis that higher than average testosterone levels are indicators of risky behaviors or disadvantaged backgrounds, both of which are associated with PTSD [52] or that acute or chronic stress might influence testosterone levels later in life. Furthermore, our results yielded small effect sizes, suggesting that testosterone may be a weak risk factor for PTSD symptoms. Fourth, as has been widely noted in other studies utilizing the UK Biobank, results may not generalize to other populations (like younger cohorts or those with health conditions) and communities (like those with lower income) [31]. In particular, because the majority of participants in this study are of European ancestry, the results of this study might not generalize as well to people with different ancestral backgrounds [52, 53]. Lastly, our comparative analysis showed a similar relationship between testosterone and other psychiatric phenotypes, including anxiety and depression, as between testosterone and PTSD symptoms. This indicates that the findings in this paper may not be limited to the relationship between testosterone and PTSD symptoms. Whether the association between testosterone and PTSD symptoms is either confounded by the association between testosterone and other psychiatric disorders or caused by the underlying shared molecular mechanisms across common disorders is outside the scope of the current study. Therefore, we recommend that future studies further disentangle this complex relationship using longitudinal datasets with multiple measures for both testosterone and psychiatric outcomes.

In sum, we attempted to better characterize the relationship between testosterone levels in both men and women and the presence of PTSD symptoms using a considerably large sample size. We found that mid-range levels of testosterone, normed separately in men and women, were associated with the lowest future PTSD symptom scores. The larger sample size further afforded interrogation of non-linear relationships and modification via BMI. Additional analysis showed that the findings in this paper may not be limited to the relationship between testosterone and PTSD symptoms, which may be due to the non-specificity of our PTSD symptom measure. Further studies exploring this relationship using longitudinal cohorts, with more detailed information and PTSD diagnostic criteria, would also be useful.

## Supplementary information

Table S1-S16

Figures S1-S7

## Data Availability

Full results from this project are available in the paper and Supplementary Materials. We are not permitted to share individual-level data from the UK Biobank, but these data are available to researchers who have received approval to access and analyze the UK Biobank data.
