# Supplementary material for: Associations between testosterone and future PTSD symptoms among middle age and older UK residents
Source: Transl Psychiatry. 2025 Aug 6;15:268. doi: 10.1038/s41398-025-03482-5 (PMC12328684; doi:10.1038/s41398-025-03482-5)
Supplement: Supplementary file 2 — Figures S1-S7 [file 41398_2025_3482_MOESM2_ESM.pptx]

## Slide 1
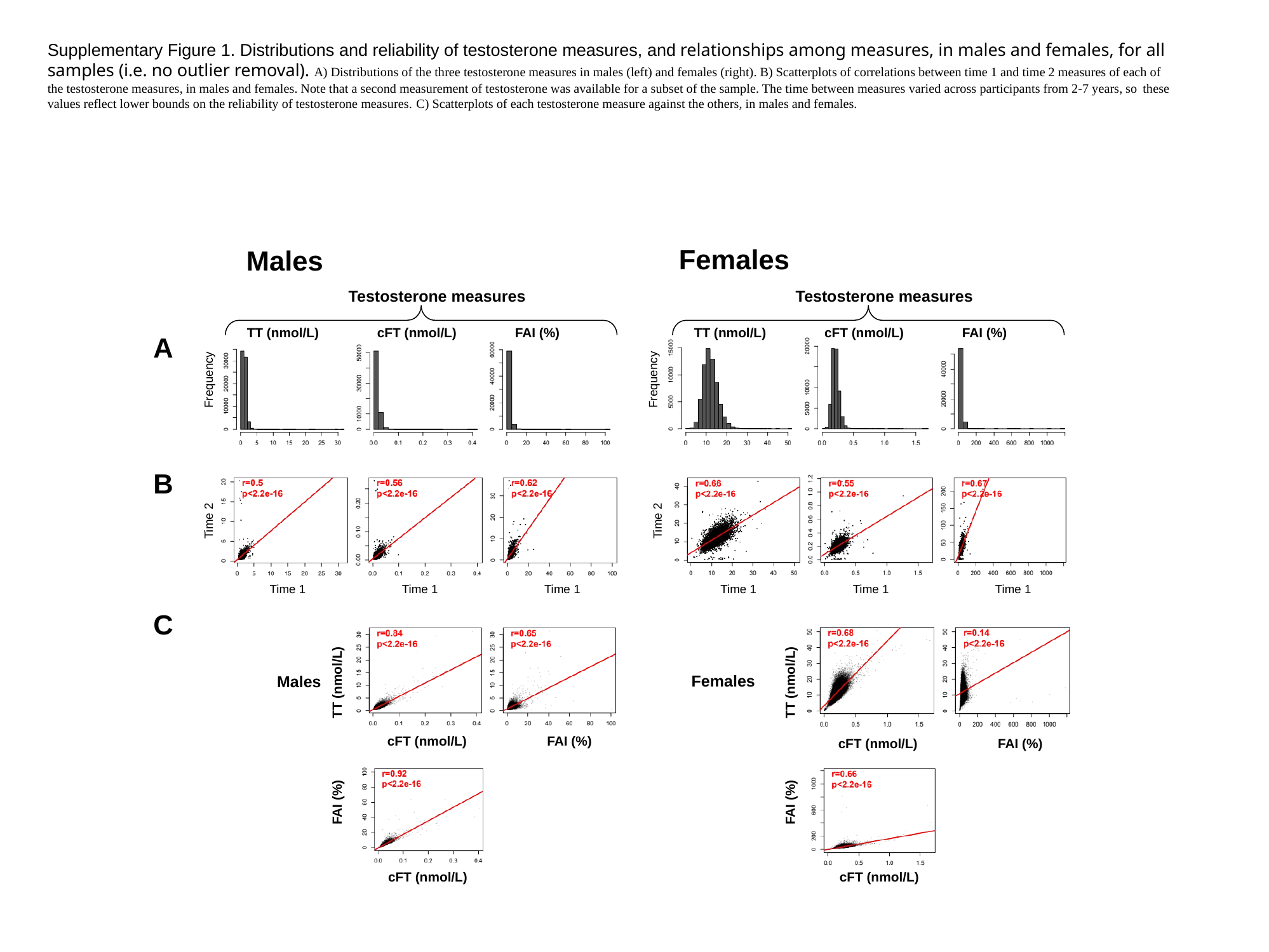

# Supplementary Figure 1. Distributions and reliability of testosterone measures, and relationships among measures, in males and females, for all samples (i.e. no outlier removal). A) Distributions of the three testosterone measures in males (left) and females (right). B) Scatterplots of correlations between time 1 and time 2 measures of each of the testosterone measures, in males and females. Note that a second measurement of testosterone was available for a subset of the sample. The time between measures varied across participants from 2-7 years, so these values reflect lower bounds on the reliability of testosterone measures. C) Scatterplots of each testosterone measure against the others, in males and females.
Females
Males
Testosterone measures
 TT (nmol/L) cFT (nmol/L) FAI (%)
Testosterone measures
 TT (nmol/L) cFT (nmol/L) FAI (%)
A
Frequency
Frequency
B
Time 2
Time 2
 Time 1 Time 1 Time 1
 Time 1 Time 1 Time 1
C
TT (nmol/L)
FAI (%)
TT (nmol/L)
FAI (%)
Females
Males
 cFT (nmol/L) FAI (%)
 cFT (nmol/L) FAI (%)
 cFT (nmol/L)
 cFT (nmol/L)

## Slide 2
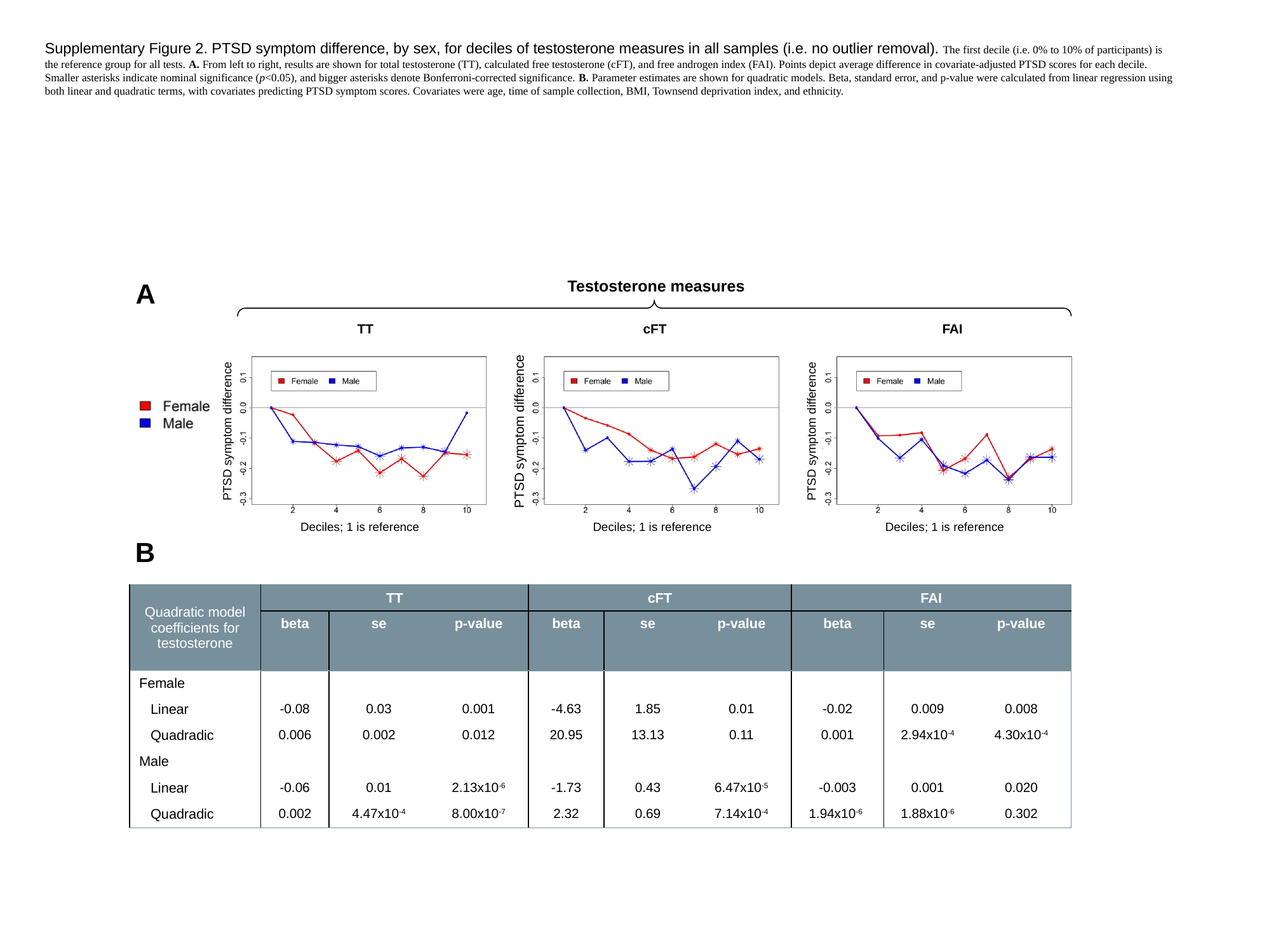

# Supplementary Figure 2. PTSD symptom difference, by sex, for deciles of testosterone measures in all samples (i.e. no outlier removal). The first decile (i.e. 0% to 10% of participants) is the reference group for all tests. A. From left to right, results are shown for total testosterone (TT), calculated free testosterone (cFT), and free androgen index (FAI). Points depict average difference in covariate-adjusted PTSD scores for each decile. Smaller asterisks indicate nominal significance (p<0.05), and bigger asterisks denote Bonferroni-corrected significance. B. Parameter estimates are shown for quadratic models. Beta, standard error, and p-value were calculated from linear regression using both linear and quadratic terms, with covariates predicting PTSD symptom scores. Covariates were age, time of sample collection, BMI, Townsend deprivation index, and ethnicity.
			Testosterone measures
 TT		 	 cFT		 FAI
A
PTSD symptom difference
PTSD symptom difference
PTSD symptom difference
 Deciles; 1 is reference		 Deciles; 1 is reference		 Deciles; 1 is reference
B
| Quadratic model coefficients for testosterone | TT | | | cFT | | | FAI | | |
| --- | --- | --- | --- | --- | --- | --- | --- | --- | --- |
| | beta | se | p-value | beta | se | p-value | beta | se | p-value |
| Female | | | | | | | | | |
| Linear | -0.08 | 0.03 | 0.001 | -4.63 | 1.85 | 0.01 | -0.02 | 0.009 | 0.008 |
| Quadradic | 0.006 | 0.002 | 0.012 | 20.95 | 13.13 | 0.11 | 0.001 | 2.94x10^-4^ | 4.30x10^-4^ |
| Male | | | | | | | | | |
| Linear | -0.06 | 0.01 | 2.13x10^-6^ | -1.73 | 0.43 | 6.47x10^-5^ | -0.003 | 0.001 | 0.020 |
| Quadradic | 0.002 | 4.47x10^-4^ | 8.00x10^-7^ | 2.32 | 0.69 | 7.14x10^-4^ | 1.94x10^-6^ | 1.88x10^-6^ | 0.302 |

## Slide 3
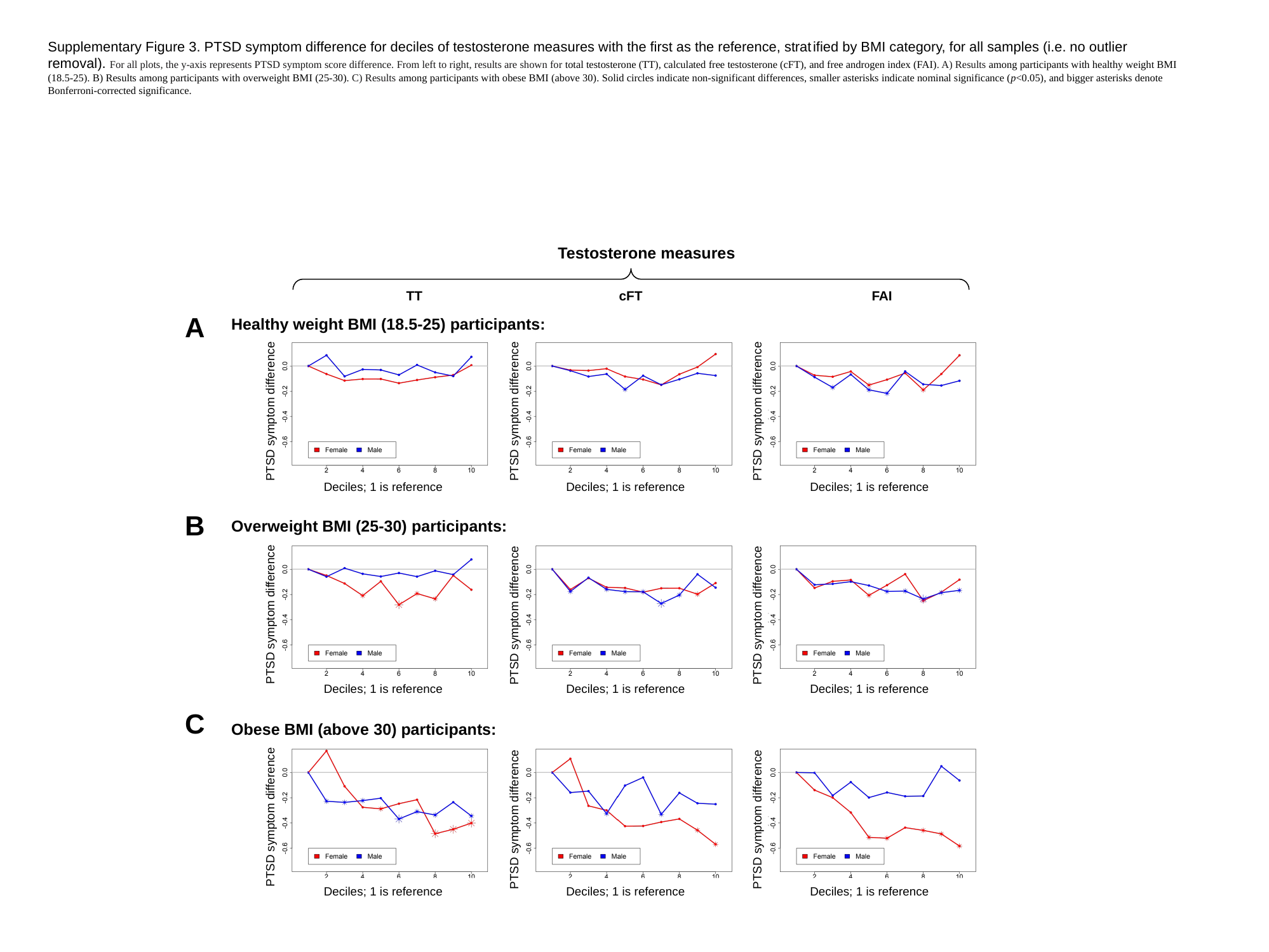

# Supplementary Figure 3. PTSD symptom difference for deciles of testosterone measures with the first as the reference, stratified by BMI category, for all samples (i.e. no outlier removal). For all plots, the y-axis represents PTSD symptom score difference. From left to right, results are shown for total testosterone (TT), calculated free testosterone (cFT), and free androgen index (FAI). A) Results among participants with healthy weight BMI (18.5-25). B) Results among participants with overweight BMI (25-30). C) Results among participants with obese BMI (above 30). Solid circles indicate non-significant differences, smaller asterisks indicate nominal significance (p<0.05), and bigger asterisks denote Bonferroni-corrected significance.
		 Testosterone measures
 TT		 cFT	 FAI
A
Healthy weight BMI (18.5-25) participants:
PTSD symptom difference
PTSD symptom difference
PTSD symptom difference
 Deciles; 1 is reference		Deciles; 1 is reference	 Deciles; 1 is reference
B
Overweight BMI (25-30) participants:
PTSD symptom difference
PTSD symptom difference
PTSD symptom difference
 Deciles; 1 is reference		Deciles; 1 is reference	 Deciles; 1 is reference
C
Obese BMI (above 30) participants:
PTSD symptom difference
PTSD symptom difference
PTSD symptom difference
 Deciles; 1 is reference		Deciles; 1 is reference	 Deciles; 1 is reference

## Slide 4
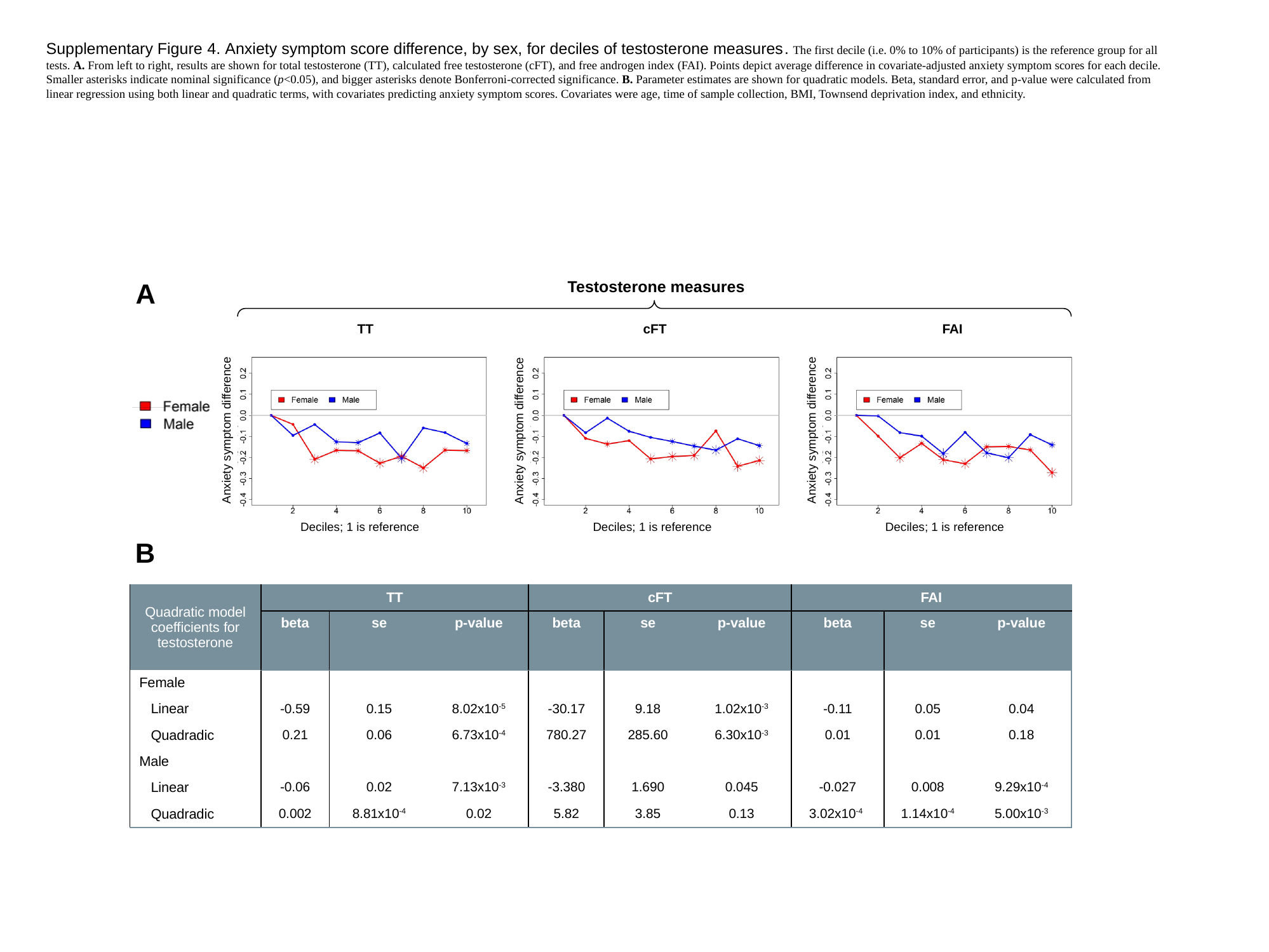

# Supplementary Figure 4. Anxiety symptom score difference, by sex, for deciles of testosterone measures. The first decile (i.e. 0% to 10% of participants) is the reference group for all tests. A. From left to right, results are shown for total testosterone (TT), calculated free testosterone (cFT), and free androgen index (FAI). Points depict average difference in covariate-adjusted anxiety symptom scores for each decile. Smaller asterisks indicate nominal significance (p<0.05), and bigger asterisks denote Bonferroni-corrected significance. B. Parameter estimates are shown for quadratic models. Beta, standard error, and p-value were calculated from linear regression using both linear and quadratic terms, with covariates predicting anxiety symptom scores. Covariates were age, time of sample collection, BMI, Townsend deprivation index, and ethnicity.
			Testosterone measures
 TT		 	 cFT		 FAI
A
Anxiety symptom difference
Anxiety symptom difference
Anxiety symptom difference
 Deciles; 1 is reference		 Deciles; 1 is reference		 Deciles; 1 is reference
B
| Quadratic model coefficients for testosterone | TT | | | cFT | | | FAI | | |
| --- | --- | --- | --- | --- | --- | --- | --- | --- | --- |
| | beta | se | p-value | beta | se | p-value | beta | se | p-value |
| Female | | | | | | | | | |
| Linear | -0.59 | 0.15 | 8.02x10^-5^ | -30.17 | 9.18 | 1.02x10^-3^ | -0.11 | 0.05 | 0.04 |
| Quadradic | 0.21 | 0.06 | 6.73x10^-4^ | 780.27 | 285.60 | 6.30x10^-3^ | 0.01 | 0.01 | 0.18 |
| Male | | | | | | | | | |
| Linear | -0.06 | 0.02 | 7.13x10^-3^ | -3.380 | 1.690 | 0.045 | -0.027 | 0.008 | 9.29x10^-4^ |
| Quadradic | 0.002 | 8.81x10^-4^ | 0.02 | 5.82 | 3.85 | 0.13 | 3.02x10^-4^ | 1.14x10^-4^ | 5.00x10^-3^ |

## Slide 5
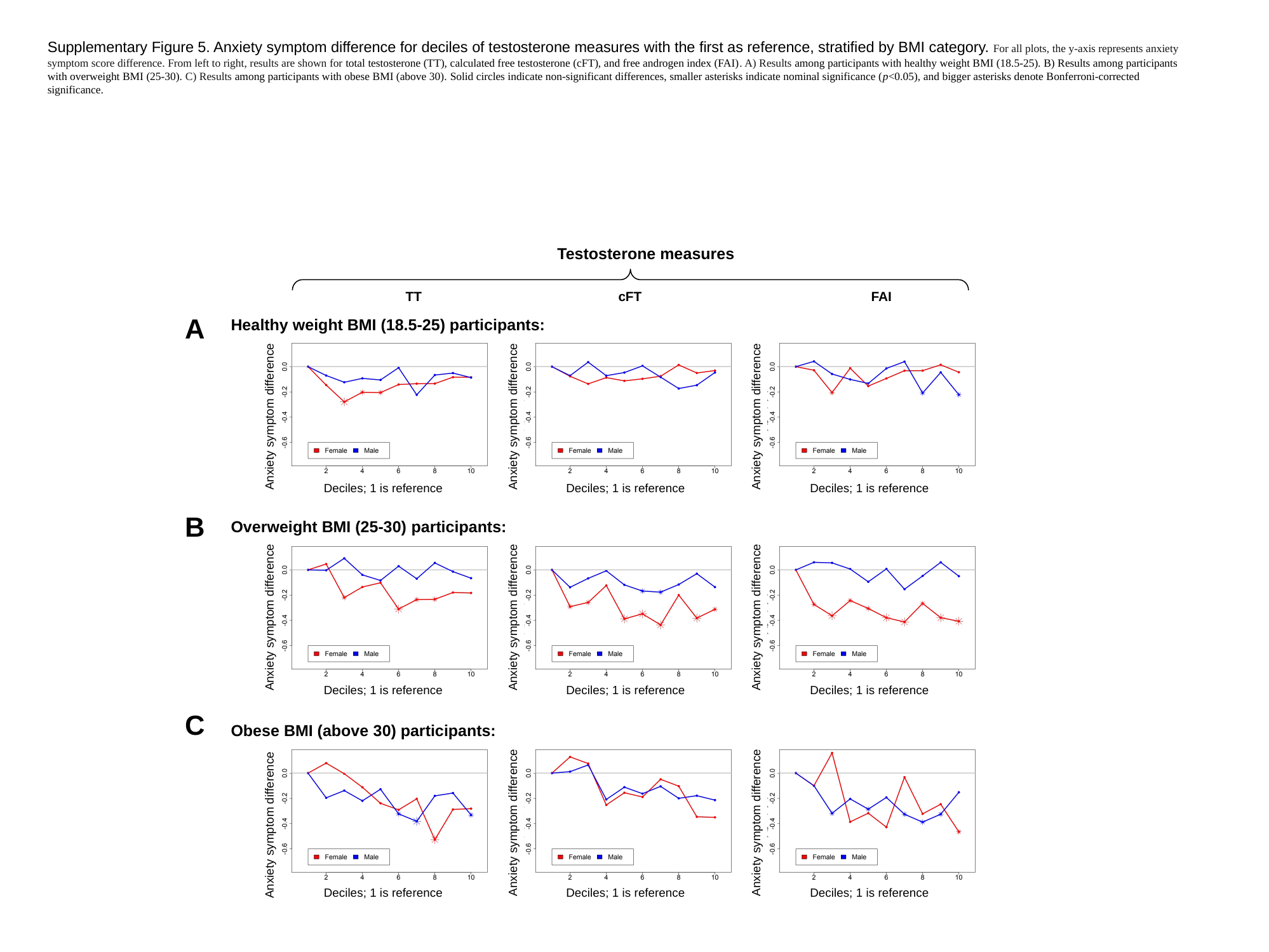

# Supplementary Figure 5. Anxiety symptom difference for deciles of testosterone measures with the first as reference, stratified by BMI category. For all plots, the y-axis represents anxiety symptom score difference. From left to right, results are shown for total testosterone (TT), calculated free testosterone (cFT), and free androgen index (FAI). A) Results among participants with healthy weight BMI (18.5-25). B) Results among participants with overweight BMI (25-30). C) Results among participants with obese BMI (above 30). Solid circles indicate non-significant differences, smaller asterisks indicate nominal significance (p<0.05), and bigger asterisks denote Bonferroni-corrected significance.
		 Testosterone measures
 TT		 cFT	 FAI
A
Healthy weight BMI (18.5-25) participants:
Anxiety symptom difference
Anxiety symptom difference
Anxiety symptom difference
 Deciles; 1 is reference		Deciles; 1 is reference	 Deciles; 1 is reference
B
Overweight BMI (25-30) participants:
Anxiety symptom difference
Anxiety symptom difference
Anxiety symptom difference
 Deciles; 1 is reference		Deciles; 1 is reference	 Deciles; 1 is reference
C
Obese BMI (above 30) participants:
Anxiety symptom difference
Anxiety symptom difference
Anxiety symptom difference
 Deciles; 1 is reference		Deciles; 1 is reference	 Deciles; 1 is reference

## Slide 6
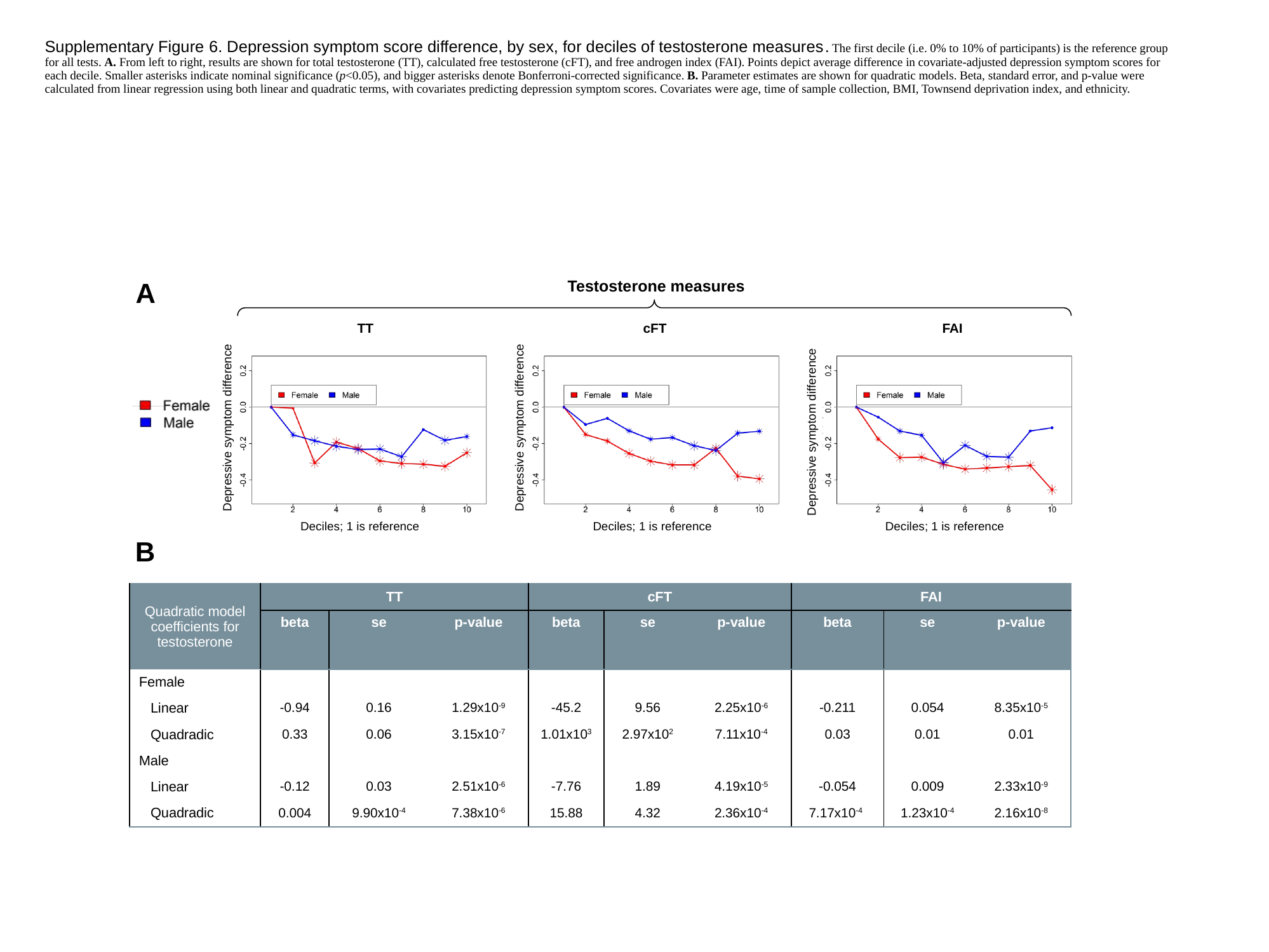

# Supplementary Figure 6. Depression symptom score difference, by sex, for deciles of testosterone measures. The first decile (i.e. 0% to 10% of participants) is the reference group for all tests. A. From left to right, results are shown for total testosterone (TT), calculated free testosterone (cFT), and free androgen index (FAI). Points depict average difference in covariate-adjusted depression symptom scores for each decile. Smaller asterisks indicate nominal significance (p<0.05), and bigger asterisks denote Bonferroni-corrected significance. B. Parameter estimates are shown for quadratic models. Beta, standard error, and p-value were calculated from linear regression using both linear and quadratic terms, with covariates predicting depression symptom scores. Covariates were age, time of sample collection, BMI, Townsend deprivation index, and ethnicity.
			Testosterone measures
 TT		 	 cFT		 FAI
A
Depressive symptom difference
Depressive symptom difference
Depressive symptom difference
 Deciles; 1 is reference		 Deciles; 1 is reference		 Deciles; 1 is reference
B
| Quadratic model coefficients for testosterone | TT | | | cFT | | | FAI | | |
| --- | --- | --- | --- | --- | --- | --- | --- | --- | --- |
| | beta | se | p-value | beta | se | p-value | beta | se | p-value |
| Female | | | | | | | | | |
| Linear | -0.94 | 0.16 | 1.29x10^-9^ | -45.2 | 9.56 | 2.25x10^-6^ | -0.211 | 0.054 | 8.35x10^-5^ |
| Quadradic | 0.33 | 0.06 | 3.15x10^-7^ | 1.01x10^3^ | 2.97x10^2^ | 7.11x10^-4^ | 0.03 | 0.01 | 0.01 |
| Male | | | | | | | | | |
| Linear | -0.12 | 0.03 | 2.51x10^-6^ | -7.76 | 1.89 | 4.19x10^-5^ | -0.054 | 0.009 | 2.33x10^-9^ |
| Quadradic | 0.004 | 9.90x10^-4^ | 7.38x10^-6^ | 15.88 | 4.32 | 2.36x10^-4^ | 7.17x10^-4^ | 1.23x10^-4^ | 2.16x10^-8^ |

## Slide 7
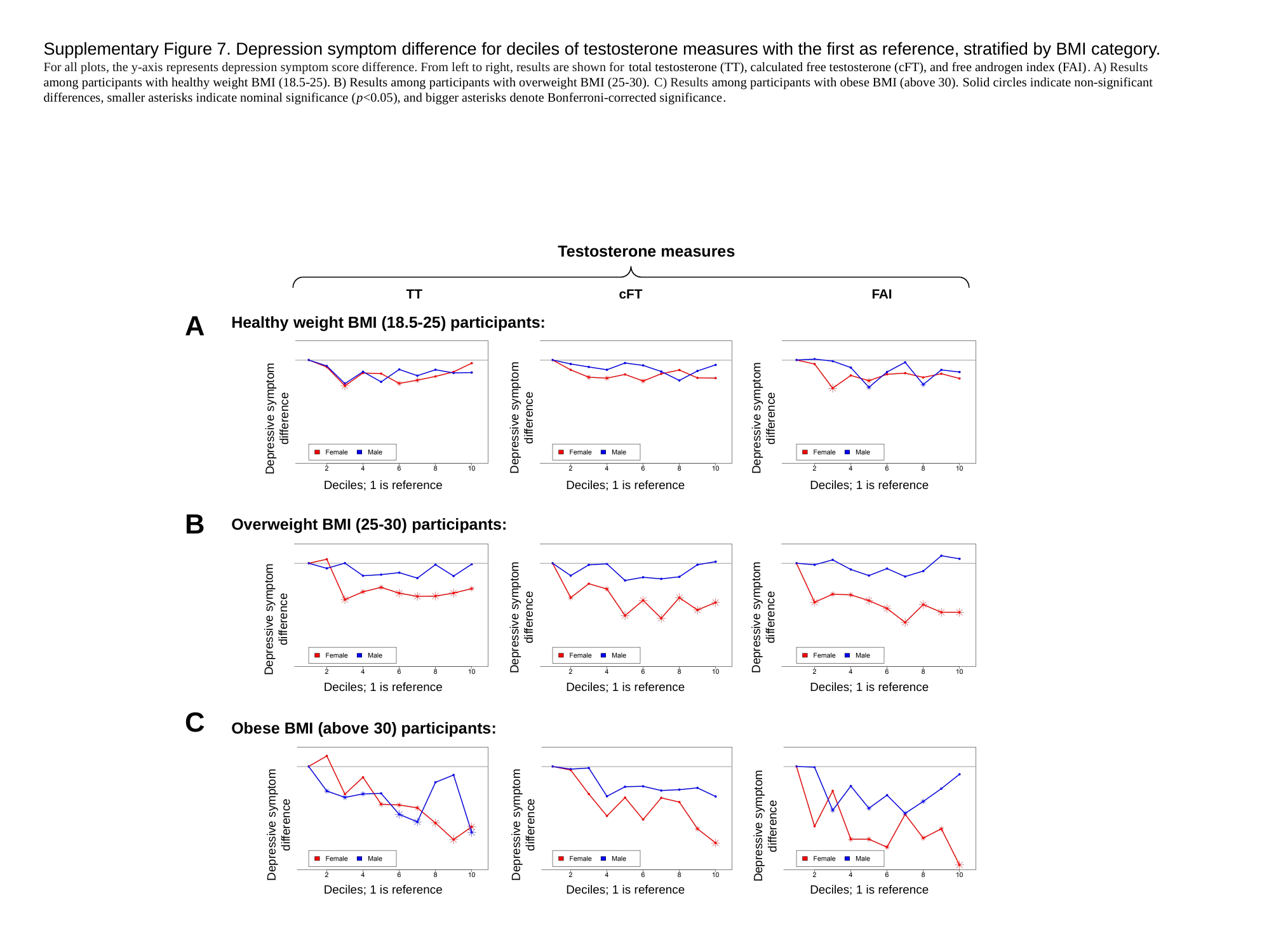

# Supplementary Figure 7. Depression symptom difference for deciles of testosterone measures with the first as reference, stratified by BMI category. For all plots, the y-axis represents depression symptom score difference. From left to right, results are shown for total testosterone (TT), calculated free testosterone (cFT), and free androgen index (FAI). A) Results among participants with healthy weight BMI (18.5-25). B) Results among participants with overweight BMI (25-30). C) Results among participants with obese BMI (above 30). Solid circles indicate non-significant differences, smaller asterisks indicate nominal significance (p<0.05), and bigger asterisks denote Bonferroni-corrected significance.
		 Testosterone measures
 TT		 cFT	 FAI
A
Healthy weight BMI (18.5-25) participants:
Depressive symptom difference
Depressive symptom difference
Depressive symptom difference
 Deciles; 1 is reference		Deciles; 1 is reference	 Deciles; 1 is reference
B
Overweight BMI (25-30) participants:
Depressive symptom difference
Depressive symptom difference
Depressive symptom difference
 Deciles; 1 is reference		Deciles; 1 is reference	 Deciles; 1 is reference
C
Obese BMI (above 30) participants:
Depressive symptom difference
Depressive symptom difference
Depressive symptom difference
 Deciles; 1 is reference		Deciles; 1 is reference	 Deciles; 1 is reference
